# Supplementary material for: Accuracy of GynTect® Methylation Markers to Detect Recurrent Disease in Patients Treated for CIN3: A Proof-of-Concept Case-Control Study
Source: Cancers (Basel). 2024 Aug 30;16(17):3022. doi: 10.3390/cancers16173022 (PMC11394525; doi:10.3390/cancers16173022)
Supplement: Supplementary file 1 [file cancers-16-03022-s001.zip › cancers-3106697-supplementary.pdf]

Supplementary Table 1. Characteristics of patients (due to rounding percentages may add up to more than 100%)

**Patient characteristics**	**Controls (*n* = 31)**	**Cases (*n* = 17)**	**Total (*n* = 48)**
Age (years), median (range)	31 (25–53)	31 (27–49)	31 (25–53)
Number of life births, *n* [%]			
None	17 (55)	9 (53)	26 (54)
One	7 (23)	6 (35)	13 (27)
More than one	7 (23)	2 (12)	9 (19)
Any contraception, *n* [%]	21 (68)	11 (65)	32 (67)
Thereof contraceptive pill	13 (62)	9 (82)	185 (64.9)
Thereof condom	4 (19)	1 (9)	5 (16)
Pregnant at study entry, *n* [%]	0 (0)	0 (0)	0 (0)
Menopause, *n* [%]	4 (13)	0 (0)	4 (8)
Smoking status, *n* [%]			
Current smoker	16 (52)	8 (47)	24 (50)
Ex-smoker	2 (6)	0 (0)	2 (4)
Non-smoker	12 (39)	9 (53)	21 (44)
Unknown	1 (3)	0 (0)	1 (2)
HPV vaccination, *n/valid* [%]	3/22 (14)	2/16 (13)	5/38 (13)
Type of primary surgery, *n* [%]			
High-frequency (HF) loop	24 (77)	10 (59)	34 (71)
Laser conisation	4 (13)	4 (24)	8 (17)
HF loop and laser conisation	3 (10)	3 (18)	6 (12)
Resection margin, *n* [%]			
Negative	22 (71)	10 (59)	32 (67)
Positive	3 (10)	4 (24)	7 (15)
Unknown	6 (20)	3 (18)	9 (19.0)
